# Anti-EGFR Antibody Plus Chemotherapy Treatment in a Patient with Synchronous Merkel Cell Carcinoma and Colorectal Cancer

**DOI:** 10.7759/cureus.12916

**Published:** 2021-01-26

**Authors:** Sara Custodio-Cabello, Luis Cabezón-Gutiérrez, Magda Palka-Kotlowska, Eduardo Oliveros Acebes, Parham Khosravi-Shahi

**Affiliations:** 1 Medical Oncology, Hospital Universitario de Torrejón, Madrid, ESP; 2 Internal Medicine, Hospital Universitario de Torrejón, Madrid, ESP; 3 Medical Oncology, Hospital General Universitario Gregorio Marañon, Madrid, ESP

**Keywords:** merkel cell carcinoma, anti-egfr, chemotherapy, synchronous tumors

## Abstract

Merkel cell carcinoma (MCC) is a rare neuroendocrine cutaneous malignancy. During early stages, surgery is the primary treatment followed by radiotherapy in patients at high risk of recurrence. Definitive radiation therapy is an alternative for patients who are not surgical candidates, reserving chemotherapy for metastatic disease. We present a case of a male patient diagnosed with MCC and stage IV colorectal cancer and we focus on the skin tumor shrinkage after specific colorectal cancer treatment.

## Introduction

Merkel cell carcinoma (MCC) usually affects Caucasians over the age of 60 [1]. There has been an increasing incidence of MCC reported over the past years, although the Spanish rate of 0.28 cases per 100.000 persons maintains lower than other European countries, the United States, and Australia [2-4]. 

High-risk factors are light skin colour, increasing age, male sex, immunosuppression, and polyomavirus infection [5]. The risk of MCC is significantly increased in patients with other malignancies, especially hematologic malignancies (multiple myeloma, chronic lymphocytic leukemia, non-Hodgkin lymphoma) and malignant melanoma [6]. 

MCC usually presents as a rapidly, growing asymptomatic solitary, firm nodule, that has a red-violet or red-blue appearance. The predominant sites are the head and neck and extremities [7]. The diagnosis of MCC is primarily based on histological and immunohistochemical features, so a biopsy should be performed in all clinically suspicious lesions.

Surgical excision with 1-2 cm margins is the preferred initial therapy of the primary tumor. Sentinel lymph node biopsy and regional lymph node dissection should be performed in case of involvement. Adjuvant radiotherapy can be discussed. The role of chemotherapy in the adjuvant setting is controversial because of the lack of survival benefit [8]. 

A minority of cases are metastatic at presentation (5%-12%) but MCC has frequent locoregional recurrences and visceral metastatic evolution with a poor prognosis associated [9]. 

Several chemotherapy regimens have been used in unresectable or metastatic MCC disease, but the impact on overall survival is unclear. Nowadays the first-line treatment option is programmed death ligand-1 (PDL‐1)/programmed death-1 (PD‐1) inhibitor-based immunotherapy (avelumab, pembrolizumab, nivolumab) that shows evidence of activity and favorable tolerability relative to chemotherapy [10-12]. Only approximately one-half of patients will persistently benefit from immune therapy, so newer approaches are required. We share our experience and the MCC durable response to colorectal therapy observed, in order to suggest further research efforts in identifying other treatment pathways.

## Case presentation

A 73-year-old Caucasian male was admitted to our hospital with acute intestinal obstruction. Computed tomography (CT) of the abdomen showed left-sided obstructive colon tumor, retroperitoneal nodes, liver metastases, and inferior vena cava thrombosis. The tumor marker carcinoembryonic antigen was elevated (308 µg/L); other tumor markers were normal. An urgent colonoscopy was rendered. Colonoscopy reported an occlusive lesion at 30 cm from the anal verge. It was biopsied and a self-expandable metal stent was placed. The biopsy reported an adenocarcinoma. No RAS gene mutation or BRAF (v-raf murine sarcoma viral oncogene homolog B1) mutation was found.

**Figure 1:**
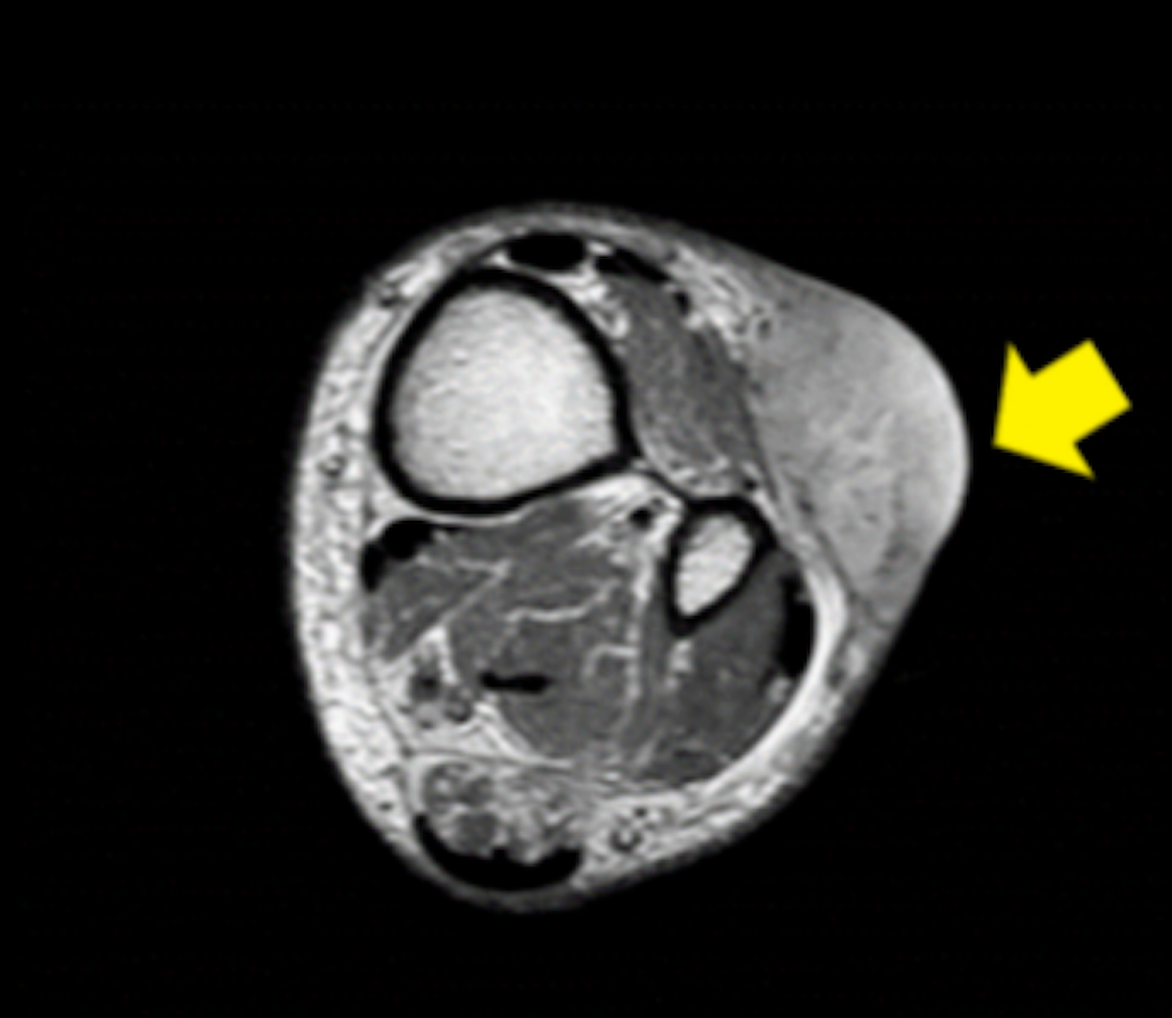
Magnetic resonance scan of Merkel cell carcinoma

During the hospital stay, the patient also complained of a painless purple-shiny bump, located in his left leg. It appeared six months ago and had been growing larger. The biopsy confirmed the diagnosis of MCC. Measurement of 4.5 cm long and involvement of long toe extensor muscle’s fascia was described in the magnetic resonance (MR) scan (Figure 1).

In the multidisciplinary tumor board, taking into account the symptoms, prognosis, and colorectal cancer spread, we decided to avoid MCC surgery and start palliative chemotherapy in order to treat metastatic colorectal cancer.

The patient was treated with bolus dose of 5-fluorouracil (5-FU) 400 mg/m^2 ^and a 46-hours of 5-FU continuous infusion of 2400 mg/m^2^, leucovorin 200 mg/m^2^, and irinotecan 180 mg/m^2 ^(FOLFIRI) - palliative chemotherapy plus the biological agent panitumumab 6 mg/kg. The administration frequency was every two weeks. 

After six cycles, body CT showed a partial response of the colon, liver, and retroperitoneal lesions. Besides, it was remarkable that the MCC was significantly reduced in size and it had changed colour (from purple to light pink).

**Figure 2:**
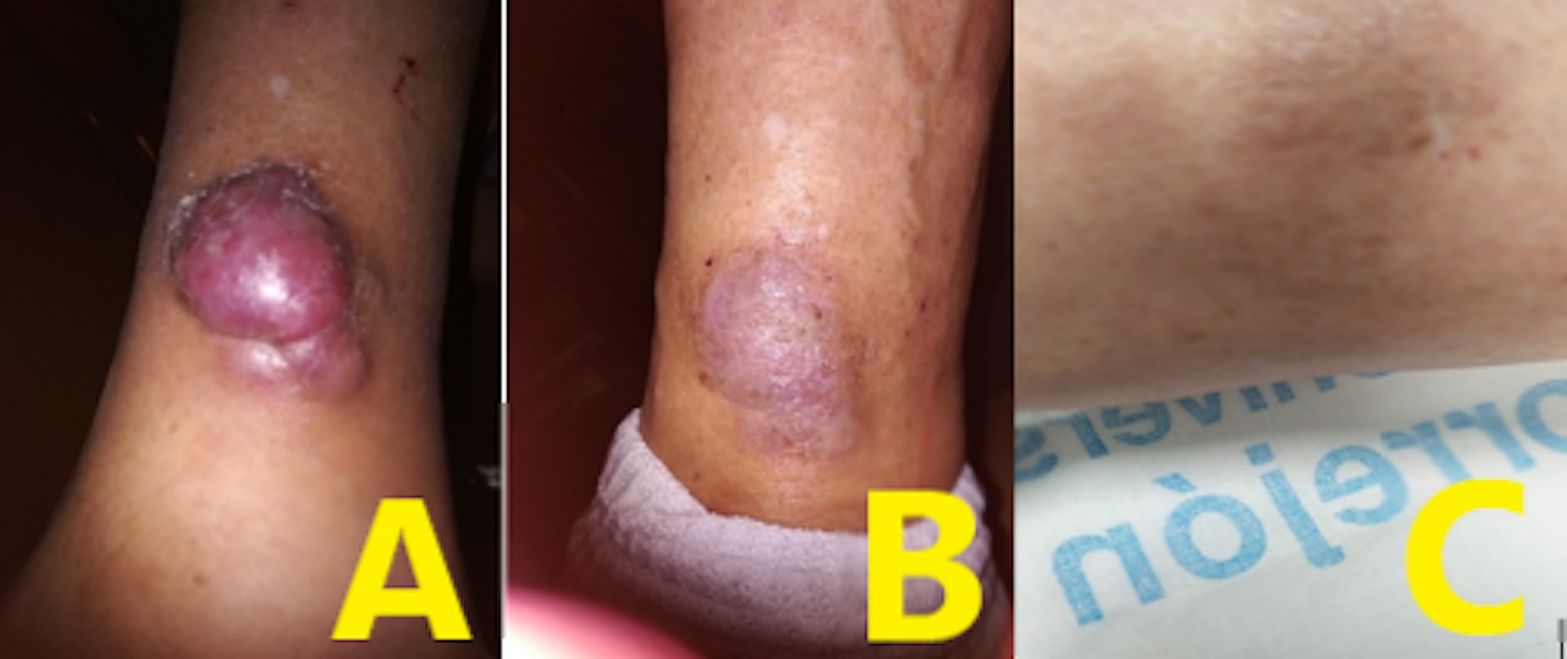
Merkel cell carcinoma evolution A: October 2019; B: May 2019; C: December 2019

The following reviews of CT described stable disease. A progression-free survival of >one year has been achieved. On the other hand, the MCC lesion continues to get smaller and flattened and it has practically disappeared (Figure 2).

## Discussion

The incidence of MCC is increased in those with hematologic malignancies (multiple myeloma, chronic lymphocytic leukemia, non-Hodgkin lymphoma) [13] and malignant melanoma [14]. It suggests the importance of immunologic factors and ultraviolet exposure in the etiology of MCC. Its associations with the salivary gland, brain, and biliary sites, other than liver and gallbladder, have also been reported. Otherwise, colorectal cancer is not significantly associated [15].

Chemotherapy is reserved for metastatic disease or palliative therapy in symptomatic patients. Classic treatment options are based on treatments for small-cell lung carcinoma due to the similar neuroendocrine properties to MCC. In most cases, these regimens combine in various ways - carboplatin, cisplatin, and etoposide, cyclophosphamide with vincristine, doxorubicin, prednisone, bleomycin, or 5-fluorouracil [16]. Although MCC is sensitive to these chemotherapy regimens, responses are not durable and are often associated with high toxicity.

Preliminary data demonstrate an early promising signal for anti-PD-L1 (avelumab) and anti-PD-1 (pembrolizumab, nivolumab) checkpoint immunotherapy in patients with metastatic MCC, providing similar response rates than those described with chemotherapy but greater durability of response.

Synchronous tumors should be treated depending on prognosis. The first tumor to treat is the one with a poorer prognosis or life-threatening ones. In our case, the patient presented with colorectal stage IV cancer that needed to start chemotherapy quickly. Local advance MCC was not our priority and we did not take it into account to choose the chemotherapy regimen. To our knowledge, anti-epidermal growth factor receptor (EGFR) treatment is not described in the literature as an option to treat MCC. We need to point out the great and long-time response observed in our patient. It is hard to say if it is related to the FOLFIRI regimen, to the anti-EGFR treatment, or to both of them.

Promising new treatment options in MCC makes it unlikely to perform clinical trials with anti-EGFR drugs to assess efficacy in this setting. Studies developed in order to find molecular targets have not found EGFR expression in MCC cells [17,18]. Anyway, small number of tumor specimens were included, so further studies will be needed to fully determine the impact of targeted therapy in MCC in vitro and in vivo, as the presence of a target does not always predict response to a targeted agent [19].

## Conclusions

MCC is a rare and aggressive skin cancer. Occasionally, it is diagnosed at the same time as other malignancies. When that occurs, the treatment choice is based on the prognosis of both tumors. Anti-PDL-1 and Anti-PD-1 checkpoint immunotherapy is the elective treatment for advanced MCC but other strategies might be explored to improve the survival, the response, and the toxicity profile of the patients. Based on other solid tumors, these treatments can include combining immune checkpoint inhibitors with other agents (conventional chemotherapy, other immunotherapy, or target therapy) or analyzing genes and biomarkers in tissue tumor to consider appropriate therapy based on the individual genome profile of each patient’s cancer.
